# Neutropenia Prediction Based on First-Cycle Blood Counts Using a FOS-3NN Classifier

**DOI:** 10.1155/2011/172615

**Published:** 2012-02-20

**Authors:** Elize A. Shirdel, Michael J. Korenberg, Yolanda Madarnas

**Affiliations:** ^1^Department of Electrical and Computer Engineering, Queen's University, Kingston, ON, Canada K7L 3N6; ^2^Division of Signaling Biology, IBM Life Sciences Discovery Centre, Toronto Medical Discovery Tower, 9-305, 101 College Street, Toronto, Ontario, Canada M5G 1L7; ^3^Departments of Oncology, Medicine, Pharmacology and Toxicology, Queen's University, Kingston, ON, Canada K7L 5P9

## Abstract

*Background*. Delivery of full doses of adjuvant chemotherapy on schedule is key to optimal breast cancer outcomes. Neutropenia is a serious complication of chemotherapy and a common barrier to this goal, leading to dose reductions or delays in treatment. While past research has observed correlations between complete blood count data and neutropenic events, a reliable method of classifying breast cancer patients into low- and high-risk groups remains elusive. *Patients and Methods*. Thirty-five patients receiving adjuvant chemotherapy for early-stage breast cancer under the care of a single oncologist are examined in this study. FOS-3NN stratifies patient risk based on complete blood count data after the first cycle of treatment. All classifications are independent of breast cancer subtype and clinical markers, with risk level determined by the kinetics of patient blood count response to the first cycle of treatment. *Results*. In an independent test set of patients unseen by FOS-3NN, 19 out of 21 patients were correctly classified (Fisher's exact test probability *P* < 0.00023 [2 tailed], Matthews' correlation coefficient +0.83). *Conclusions*. We have developed a model that accurately predicts neutropenic events in a population treated with adjuvant chemotherapy in the first cycle of a 6-cycle treatment.

## 1. Introduction

Maintenance of dose intensity in adjuvant (curative) chemotherapy is associated with improved outcome in early-stage breast cancer [1, 2]. Myelosuppression is the main dose-limiting factor of cytotoxic chemotherapy and a barrier to maintenance of dose intensity. Retrospective data from a very mature study of adjuvant chemotherapy for early-stage breast cancer suggested that patients receiving less than 65% of the intended dose did not benefit from adjuvant chemotherapy, highlighting the importance of dose intensity maintenance throughout treatment [3]. Neutropenia is the most common type of myelosuppression and often prompts dose reductions or delays. Use of hematopoietic growth factors can reduce the incidence, severity, and duration of established neutropenia. However, these agents can cause bone pain, fever and require administration by subcutaneous injection over several consecutive days. They are also costly, and not all chemotherapy regimens carry the same risk of neutropenia, thus not warranting their use for all patients preemptively [4]. However, Chang does note that there would be a marked benefit in being able to identify high-risk patients prior to beginning chemotherapy in order to rationally dispense growth factor support and avoid the occurrence of both dose reduction and delay [5]. Given the cost of these agents, there is also an economic argument to enhanced patient selection that would enable more rational resource allocation [6].

Many papers cite the need for a tool to identify high-risk patients among those undergoing adjuvant chemotherapy for early-stage breast cancer [5–7]. Several authors have demonstrated correlations between risk groups and blood count data, in various malignancies for specific regimens, but none are able to produce a broad and robust predictor that transcends tumour subtype and treatment regimen to distinguish high risk patients from low risk patients in breast cancer [8–21]. This paper presents a nonlinear model to predict which patients will be at high-risk for a neutropenic event based on information available in the first cycle of a 6-cycle adjuvant chemotherapy regimen for early-stage breast cancer. The model has shown high accuracy (>90% overall) over independent test sets and was derived using fast orthogonal search (FOS) [22].

FOS was first described as a robust and efficient method for approximating time series data and nonlinear systems of unknown structure. FOS constructs a concise model of the form

(1) $$y\left(n\right)=\overset{M}{\underset{m=0}{\sum }}a_{m}p_{m}\left(n\right)+e\left(n\right),$$

where *y*(*n*) is the time series data or the system output to be approximated, the *p*_*m*_(*n*) are the model terms selected from a set of candidates, and *e*(*n*) is the model error. In the present study, *y*(*n*) equals 1 for each patient *n* in our training set subsequently suffering a neutropenic event and equals −1 otherwise, and the candidates are the first-cycle blood counts and all possible second-order crossproducts thereof. The selected *p*_*m*_(*n*) are the critical terms that will be used subsequently to predict neutropenia for new patients. Since FOS exploits the implicit computation of orthogonalized basis functions of the search terms, without actually computing the orthogonalized functions themselves, FOS is an extremely rapid method to model systems. For each iteration, FOS selects the basis function that maximizes the reduction in mean-squared error and adds it to the model. Iterations cease when the addition of model terms no longer reduces the MSE significantly, and then the coefficients *a*_*m*_ are calculated. FOS has proven to be highly effective at selecting predictive model terms and recently has been applied in uses as varied as indoor WiFi positioning [23] and predicting heat-related emergency room visits [24]. Coupled with a 3-nearest neighbour classifier based on the FOS-selected blood markers, FOS-3NN is able to identify patients at high and low risk for neutropenia early in the course of a chemotherapy regimen.

## 2. Materials and Methods

### 2.1. Patients

This study was approved by the institutional ethics board. Patient data used to train and test the models in this retrospective study were drawn from a single clinical oncology practice belonging to one of the authors (Y. Madarnas) at the Cancer Centre of Southeastern Ontario (CCSEO). The files of all women who initiated adjuvant chemotherapy for early-stage breast cancer between January 1, 2001 and December 31, 2003 were examined by two of the authors (Y. Madarns, E. A. Shirdel). Follow-up period for this study began at day 1 of chemotherapy treatment and ended on the last day of the last cycle. We included only six-cycle adjuvant chemotherapy regimens (CEF—cyclophosphamide/epirubicin/fluorouracil; CAF—cyclophosphamide/adriamycin/fluorouracil; CMF—cyclophosphamide/methotrexate/fluorouracil). Treatments were assigned through a rule-based system as per the standard of care at the time. This cohort was further restricted to women receiving their entire course of treatment at the CCSEO, since some patients completed their treatment at a satellite clinic. This selection process yielded a cohort of 35 patients from a single clinical practice over a 36-month period, all managed in a homogenous fashion and treated with a regimen of equal duration. No preemptive growth factor support was used during the entire time. Growth factors were used only as secondary prophylaxis once a neutropenic complication had occurred. Patient characteristics shown in Tables 1(a) and 1(b) show the breakdown of patient treatment characteristics.

### 2.2. Data Collection and Processing

The data collected included, but were not limited to, a baseline count taken on day 0 or prior to the commencement of treatment, day 7, and day 28 of the first cycle of treatment. Blood count data were collected similarly for subsequent treatment cycles including those that were delayed for any reason, such as reasons grounded in clinician decisions based on avoidance of neutropenia and related complications. Reasons for delays, as well as timing and details of events occurring during treatment, were recorded.

The required information was abstracted from the complete blood count data for each patient obtained from blood tests on days that the patient was in the clinic. Biomarkers examined included absolute neutrophil count (ANC), white blood cell count (WBC), hemoglobin levels (HGB), and platelet levels (PLT) which are listed in Table 2. To equally weight different blood markers, values were normalized to fall within a range of 0.02 to 13.5.

In this study, it was crucial that all patients have the same data points available for analysis. Hence, any patient missing counts on vital days of treatment was excluded from the study. Fortunately, from the original 36-patient dataset, only one patient had incomplete recorded data and was excluded from this study.

### 2.3. Outcome Events

The primary goal of this research was to identify reliable predictors of neutropenia that are available to physicians during the onset of chemotherapy. To accomplish this, we built a nonlinear model based on CBC data available in the first cycle of a six-cycle chemotherapy regimen. Using the first-cycle data, the model was trained to classify patients into two risk groups: patients at high risk for developing a neutropenic event over the course of the treatment and patients at low risk. Patients were retrospectively classified into these groups based on knowledge of their treatment outcomes. Endpoints of interest and risk group assignation are similar to those used by Chang [5] and are presented in Table 3. Should a patient have characteristics falling into both the low- and high-risk categories, the patient is classified at the higher-risk level.

## 3. Statistical Analysis

### 3.1. Model Identification: Fast Orthogonal Search

**Figure 1:**
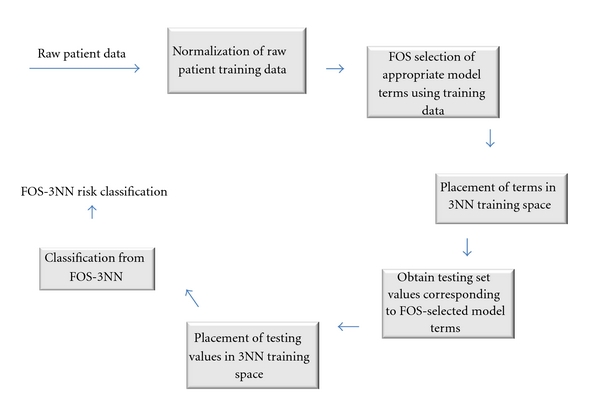
Pipeline of FOS-3NN sequence.

FOS-3NN combines fast orthogonal search (FOS) [22] with a 3-nearest neighbor classifier [25]. In the first stage of the model, FOS is used to identify input terms relevant to clinical outcome (high versus low risk). This stage narrows down a set of 90 first- and second-order cross-product terms, based on blood counts, to select the 11 terms that have the strongest predictive power. FOS is a nonlinear modeling technique that views the problem at hand as a “black-box” scenario and converts input blood count terms into prediction class variables. The known first-order inputs to the system under study here were the blood counts during the first cycle of treatment. In training, patients at high risk were assigned an outcome value of +1, and patients at low risk were assigned an outcome value of −1. The strength of FOS when used in this manner is to determine, from the given candidate set of blood markers, those terms that are most highly predictive of the output values of the system under study, thus identifying key early predictors of neutropenia. These predictors are a significant contribution of this paper; their effectiveness is demonstrated here with a 3NN classifier, but they can also be used with other classifiers. The FOS-3NN pipeline is shown in Figure 1.

Once FOS determined the optimal model terms for classification across all patients in a training set, their values were mapped as the coordinates of vectors for the training set in an 11-dimensional nearest neighbor classifier. Optimal model terms are shown in Table 4.

The FOS model in this work was trained on 14 patients undergoing chemotherapy. In all, 12 blood count values were used per patient. The 12 terms along with their 78 second-order cross-products (including squares) formed the candidate set from which the terms most indicative of impending neutropenia were chosen.

### 3.2. Model Validation

It is important to note that the model validation in this experiment was done on two different sets of data, which are both completely independent of the training dataset. The first testing set consisted of 14 patients evenly split between high and low risk. Using the FOS-3NN method, all of the 14 testing set patients were classified based on their proximity to the training patients by majority vote of the three nearest neighbours. A further independent validation set of 7 patients was also tested. These seven patients consisted of 4 high-risk patients and 3 low-risk patients. This time, the 11-space nearest neighbor classifier was filled with all of the first 28 patients, each situated at the coordinates of their pertinent classifying terms as established by FOS-3NN (Table 4). This last validation set was done to examine if there seemed to be any advantage to filling the NN classifier with more training points than the original 14 that were used with the first validation set. It also further establishes the robustness of the model and its ability to transcend training sets to make accurate classifications on data never before encountered.

## 4. Results

The FOS-3NN classifier correctly classified 19 of the 21 patients in these two sets combined. None of the low-risk and only 2 of the high-risk patients were misclassified. Fisher's exact test probability is *P* < 0.00019 (1 tailed) and *P* < 0.00023 (2 tailed). Fisher's exact test was conservatively used due to the small sample sizes in this study and is similar to the chi-square statistic for larger studies. The corresponding Matthews' correlation coefficient is phi = +0.83. Matthews' correlation coefficient is used for binary-valued classifications and ranges from +1 for a perfect prediction set to −1 for a completely incorrect prediction set. As an added test, the model was rebuilt switching the initial 14-patient testing and training sets but leaving the independent 7 patients as part of the testing procedure. Identifying the optimal classification terms on this new training set resulted in 11 chosen terms, 3 of which were also chosen the first time this model was built based on the original training set. With these 11 chosen terms in the 3-NN classifier, on the 21 patients reserved for testing 17 out of 21 were correctly classified. Four of the 10 low-risk patients were misclassified and 0 out of the 11 high-risk patients were misclassified resulting in Fisher's exact test probability of *P* < 0.0039 (1 or 2 tailed) and Matthews' correlation coefficient of phi = +0.66. Recalling that all of these classifications were made based on blood marker values available in the first 4 weeks of a 24-week chemotherapy regimen, we can see just how clinically valuable this type of risk prediction can be.

**Figure 2:**
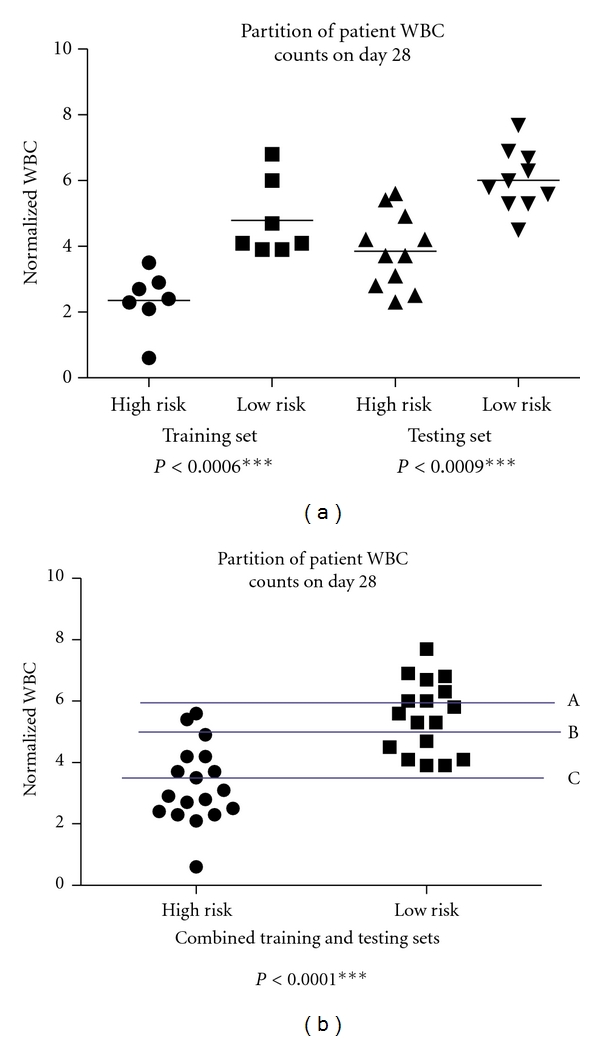
(a) Although *t-*tests show high significance in many first-order terms, the dotplots above underscore that a significant difference in the WBC counts on day 28 between high- and low-risk groups—resulting in a highly significant *P* value—is not sufficient to partition the risk groups. (b) Examining the entire cohort, it can be seen that slicing the populations by neither line A (10 patients misclassified), line B (8 patients misclassified), nor line C (7 patients misclassified) will provide good results.. Clearly, we need a more complex model to stratify this population.

In creating predictive models for clinical applications such as the prediction of neutropenia, it is critically important to understand the enormous difference between a clinically correlated variable and a model of predictive value.  Table 5 shows all first-order CBC values from which (along with their cross-products) FOS selected the predictors. We note that there are several highly significant variables capable of distinguishing between the two risk groups by a student's *t-*test. Similarly, Table 6 shows the hazard ratios for all first-order variables. According to these tables, there are several first-order terms that should be useful as classifiers of risk, including PLT0, WBC7, PLT7, ANC7, WBC28, HGB28, and ANC28.  Figure 2(a) plots both the training and testing set data for WBC counts on day 28—a variable with a highly significant *P* value between the low- and high-risk groups.  Figure 2(b) plots the same variable attempting to partition the combined training and testing sets. It becomes clear in Figure 2(b) that although the 2 risk groups appear quite different when stratified by WBC28 values, it still remains difficult to classify the patients outright. Neither a partition at line A (which misclassifies 10 low-risk patients), line B (which misclassifies 2 high- and 6 low-risk patients), or line C (which misclassifies 7 high-risk patients) does a good job at dividing the risk groups. Hence, a classification based on WBC28 alone—a clearly significant first-order term—will provide poor prognostic value.

## 5. Discussion

FOS has been used elsewhere for feature selection, predicting heat-related emergency department visits, where FOS searched about 140,000 candidate terms to find within minutes a concise 3-term model, each term a cross-product of multiple predictors [24]. While the role of FOS in feature selection has similarity to other feature selection methods such as principal component analysis (PCA) and partial least squares, there are important differences. For example, FOS finds features that have physical meaning, whereas PCA finds a few linear combinations (eigenvectors) of all the candidates, and these linear combinations do not have physical meaning. In a recent application to WiFi indoor positioning, FOS was significantly faster, and also more accurate, than PCA [23].

In our study, all but one of the selected terms involved nonobvious cross-product combinations of certain blood count measures. Although the effectiveness of these terms in predicting neutropenia was demonstrated by using them in a three-nearest neighbour (3-NN) classifier, they could also be incorporated into many other classifiers such as weighted voting [26], support vector machines [27], and IBM SPLASH [28].

**Figure 3:**
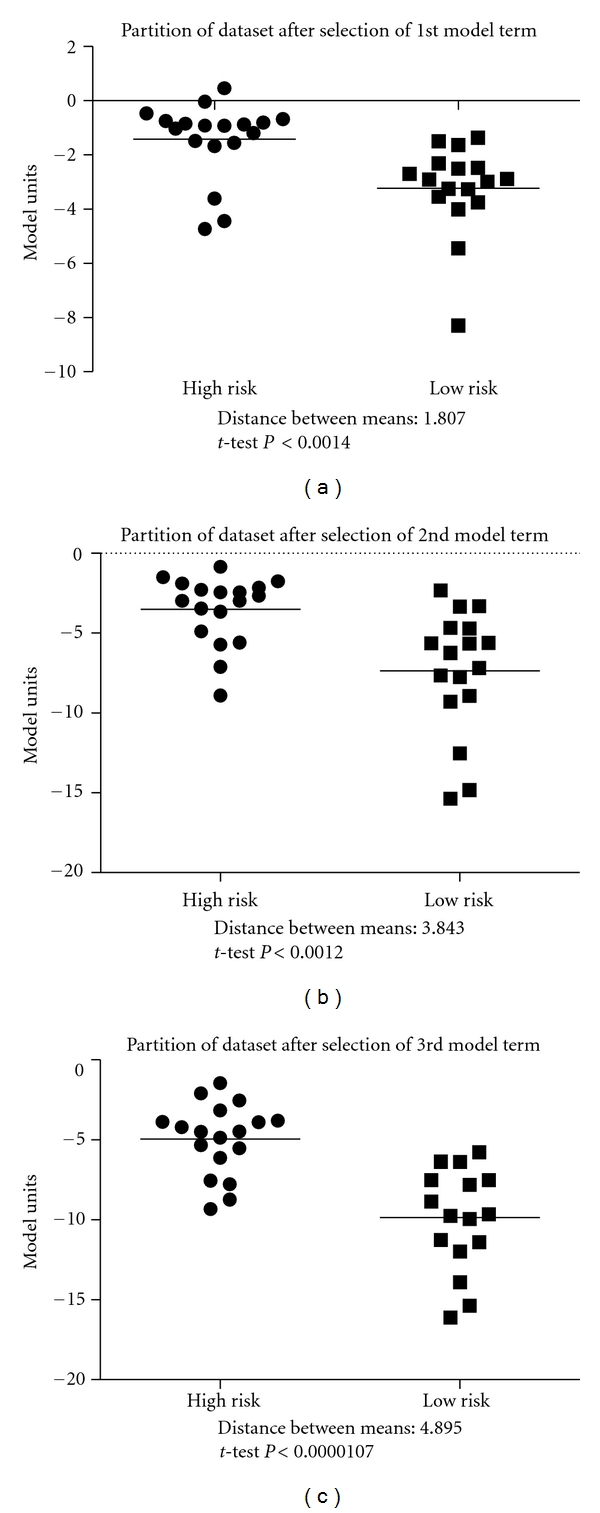
(a)–(c) show a 2D representation of the chronological improvement of the partitioning of data through the addition of model terms. This improvement can be measured by the distance between the means as indicated below the graphs.

**Figure 4:**
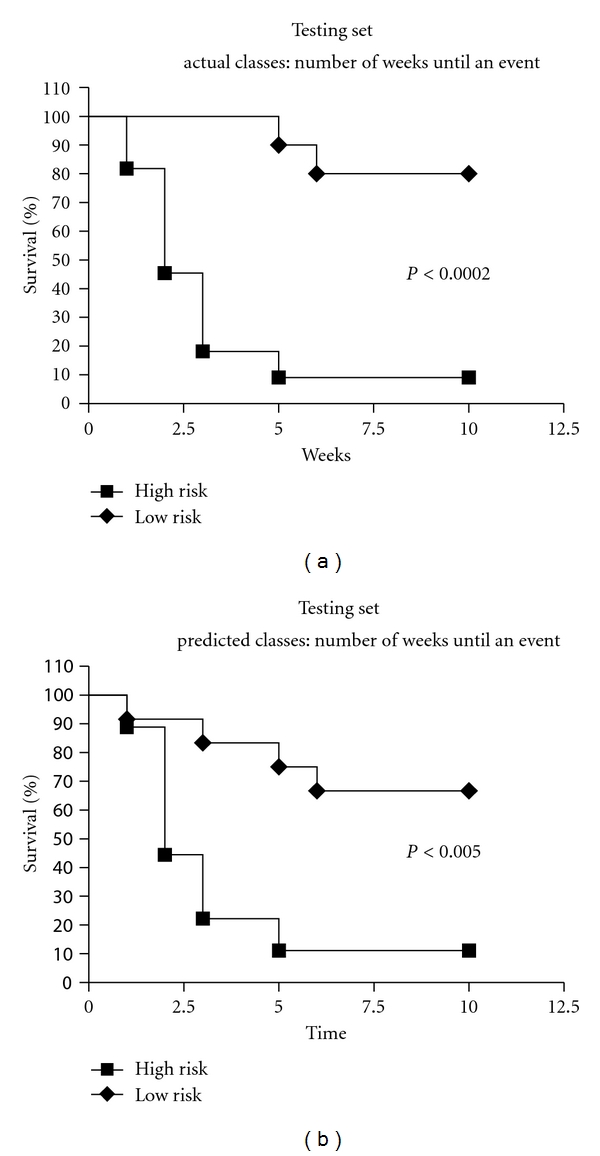
The testing set survival curves for the actual patient population (a) and the predicted classes (b).

Figures 3(a)–3(c) show a 2D representation of the effect of adding more terms to the FOS model. Not only does the intergroup distance increase with additional model terms, the partitioning line (not pictured) between the two groups grows more complex and nonlinear in nature. Since many datasets and relationships are nonlinear in nature, FOS-3NN is an appropriate model due to its adaptability to provide a better descriptor of the differences between the groups at hand.  Figure 4 compares the Kaplan-Meier curves for the actual high- and low-risk groups and the predicted risk groups in terms of patient survival to the first event during treatment over the testing set [29].

In the present work, FOS has been used for feature selection. Table 4 listing the 11 “optimal” terms found by FOS is important because these terms have been shown here to be good predictors of neutropenia when tested on an independent set and appear to have clinical value. These terms in particular should be tested in the future on larger novel sets. If alternatively we had used cross-validation or leave-one-out testing, then one set of features would not have been shown to be effective on an independent test set. Instead, 35 different concise sets of features would have been found, each set tested on only one held-out case, while our present approach has demonstrated the effectiveness of the same set of features over an independent set. One contribution of our paper is this set of 11 features, 10 of which are cross-product terms that probably would not be obvious to clinicians, and these 11 terms can now be used in a 3NN classifier or in other classifiers by other investigators without needing any knowledge of FOS. We do not claim that 3NN is essential to be used with these FOS-found features, but very good accuracy was obtained with 3NN.

Clearly, there is much information to be harnessed and interpreted from the early kinetics of blood markers in chemotherapy regimens. FOS-3NN exploits powerful characteristics of 2 classification schemes. Fast orthogonal search allows efficient examination of the 90-member candidate set and the selection of relevant model terms. The strength of the 3-nearest neighbour classifier lies in the high correlation between the group classes and their member locations in the selected feature space. Even one poor choice of coordinate in an 11-vector training point could destroy the virtue of the other characteristics since the NN classifier employs a distance metric, which can be greatly influenced by just one uncorrelated feature. This could drastically skew the output of the cascaded 3-NN classifier and significantly degrade the accuracy of the technique. Further studies with larger datasets are needed to replicate this work on a separate and larger dataset to confirm the innate value of CBC count data in neutropenic prediction.

## 6. Conclusions

Here, we lay the groundwork for a tool that might be applied in the future to prospectively identify patients at high risk for neutropenia. Many authors have observed that incorporating a model such as the one that this paper presents into clinical practice would allow the early identification of high-risk patients to target for preventative interventions and would provide a cost-effective way to distribute expensive resources. There is little doubt that many nonlinear models will surface in future biological signaling prediction work. This paper gives us a glimpse of the clinical utility of a nonlinear model able to determine risk status for neutropenia based on early blood count data.

## Figures and Tables

**Table:** (a)

Group	Median age at first chemotherapy treatment
Training set	46.79
Testing set	44.8
Validation set	56.19
*Entire population *	**46.34**

Group	BMI at first chemotherapy treatment

Training set	24.29
Testing set	25.7
Validation set	25.86
*Entire population *	**25.71 **

**Table:** (b)

	Training set	Testing set	Validation set	Entire patient population
	*N* = 14	*N* = 14	*N* = 7	

Radiation				
Concurrent	9 (64.3%)	0 (0%)	0 (0%)	9 (25.7%)
Chemotherapy				
CEF	0 (0%)	12 (85.7%)	2 (28.6%)	14 (40%)
CAF	0 (0%)	1 (7.1%)	5 (71.4%)	6 (17.1%)
CMF	14 (100%)	1 (7.1%)	0 (0%)	15 (42.9%)
Risk				
High	7 (50%)	7 (50%)	4 (57.1%)	18 (51.4%)
low	7 (50%)	7 (50%)	3 (42.9%)	17 (48.6%)

*Note that percentages may not add to 100% due to rounding.

**Table 2:** First-order candidate model terms.

Potential model terms	
Height, weight, BMI, age, WBC0, HGB0, PLT0, ANC0, WBC7, HGB7, PLT7, ANC7, WBC28, HGB28, PLT28, ANC28	

**Table 3:** Patient classification scheme.

Characteristics of high-risk patients	Characteristics of low-risk patients
Any hospitalization	No event
3 or more delays in treatment	Delay beyond cycle 3
Any delay beyond 40 days	
Delay after the first treatment	
No treatment on day 7 in any of the first 3 cycles	
Dose reduction in first 3 cycles	

**Table 4:** Model terms as selected by FOS-3NN.

Optimal model terms	
PLT28*ANC28, ANC0*ANC0, ANC0, ANC0*ANC7, ANC7*HGB28, HGB7*PLT7, HGB0*ANC7, ANC0*WBC28, ANC7*ANC28, ANC0*ANC28, PLT7*ANC7	

**Table 5:** *t*-test for first-order blood count variables based on stratification into high- and low-risk groups.

First order	Training			Testing		
Day 0	White blood cell count	0.3532		White blood cell count	0.0050	***
	Hemoglobin count	0.3090		Hemoglobin count	0.8936	
	Platelet count	0.4773		Platelet count	0.5421	
	Absolute neutrophil count	0.4542		Absolute neutrophil count	0.0057	***

Day 7	White blood cell count	0.0756	***	White blood cell count	0.0152	***
	Hemoglobin count	0.0771		Hemoglobin count	0.5098	
	Platelet count	0.1962		Platelet count	0.8782	
	Absolute neutrophil count	0.0501	***	Absolute neutrophil count	0.0324	***

Day 28	White blood cell count	0.0010	***	White blood cell count	0.0001	***
	Hemoglobin count	0.0050	***	Hemoglobin count	0.4487	
	Platelet count	0.3107		Platelet count	0.9300	
	Absolute neutrophil count	0.0030	***	Absolute neutrophil count	0.0035	***

***Indicates t-test significance.

**Table 6:** Hazard ratios and *P* values for first-order variables.

Variable	Hazard ratio (range)	*P* value
Height	1.65334 (0.547–4.996)	0.37
Weight	0.53505 (0.143–2)	0.35
BMI	4.29314 (0.143–129.226)	0.4
Age	1.02712 (0.944–1.118)	0.54
WBC0	2.42659 (0.486–12.114)	0.28
HGB0	3.57073 (0.138–92.662)	0.44
PLT0	0.18231 (0.0508–0.654)	0.009
ANC0	0.38745 (0.0713–2.105)	0.27
WBC7	0.58236 (0.0892–3.802)	0.57
HGB7	0.00197 (0.00000174–2.226)	0.082
PLT7	5.62738 (1.41–22.451)	0.014
ANC7	0.63501 (0.0652–6.184)	0.7
WBC28	0.03496 (0.00367–0.333)	0.0035
HGB28	3.82722 (0.0379–386.047)	0.57
PLT28	2.90312 (1.15–7.334)	0.024
ANC28	10.86188 (0.779–151.382)	0.076
